# Berberine Combined with Triple Therapy versus Triple Therapy for* Helicobacter pylori* Eradication: A Meta-Analysis of Randomized Controlled Trials

**DOI:** 10.1155/2018/8716910

**Published:** 2018-10-04

**Authors:** Xiaotao Jiang, Chenguang Jiang, Cihui Huang, Guoming Chen, Kailin Jiang, Baoyi Huang, Fengbin Liu

**Affiliations:** ^1^Guangzhou University of Chinese Medicine, Guangzhou, China; ^2^Department of Spleen and Stomach, First Affiliated Hospital of Guangzhou University of Chinese Medicine, Guangzhou, China

## Abstract

**Objective:**

To assess the effects and safety of berberine combined with triple therapy on* Helicobacter pylori* (*H. pylori*) eradication in adults.

**Methods:**

PubMed, MEDLINE, EMBASE, Cochrane Library, and Chinese databases including China National Knowledge Infrastructure (CNKI), Wanfang data, Chinese Technology Journal Full-text Database (VIP), and China biomedical literature database (CBM) were searched to obtain the eligible studies published up to October 10, 2017. The primary outcome was eradication rate of* H. pylori*. The secondary outcome was incidence of adverse effects. Data analysis was conducted by RevMan5.2 and Stata V.9.0 software. Trial sequential analysis (TSA) was performed to assess the risk of random error and the validity of conclusion with TSA program version 0.9 beta.

**Results:**

The meta-analysis results indicated berberine combined with triple therapy could improve the eradication rates of* H. pylori* (urea breath test subgroup: RR=1.18, 95%CI=(1.12,1.24), *P* < 0.00001, biopsy subgroup: RR=1.23, 95%CI=(1.13,1.34), *P* < 0.00001) and reduce the total occurrence of adverse effects (OR=0.59, 95%CI(0.46, 0.75), *P* < 0.0001) when compared with only using triple therapy. Besides, the incidence of nausea (OR=0.59, 95%CI(0.41, 0.86), *P* < 0.05) and diarrhea (OR=0.41, 95%CI(0.24, 0.71) was remarkably lower in experimental group while that of abdominal distention (OR=0.64, 95%CI(0.40,1.04), *P* > 0.05) and vomiting (OR=0.65, 95%CI(0.37, 1.15), *P* > 0.05) had no significant change. TSA of* H. pylori* eradication rates and adverse effects incidence illustrated that the cumulative value of Z-curve went across the conventional boundary value, trial sequential monitoring boundary for benefit, and required information size, suggesting the results were stable.

**Conclusion:**

Evidence from meta-analysis suggested that berberine combined with triple therapy can be an option for increasing* H. pylori* eradication rates and reducing overall therapy-related adverse effects incidence, particularly nausea and diarrhea, whereas more randomized controlled trials designed according to CONSORT statement are demanded to support the efficacy in further studies.

## 1. Introduction

### 1.1. Introduction of Condition

*Helicobacter pylori (H. pylori)* is a spiral, microaerophilic, gram-negative bacteria associated exclusively with gastric mucosal cells [1]. It could cause damage to the cells and lead to the clinical consequences ranging from asymptomatic gastritis to peptic ulceration and gastric malignancy [2, 3], which has been classified as class I carcinogen by WHO [4]. It was reported that more than 50% of the world's population was infected with* H. pylori* [5]. At present, the clinical treatment is a triple regimen, that is, proton pump inhibitors or bismuth combined with two antibiotics in order to achieve the inhibition of gastric acid secretion, protection of gastric mucosa, and resistance to* H. pylori*. However, with the widespread application of this therapy,* H. pylori* resistance to metronidazole, clarithromycin, and other antibiotics is gradually increasing, causing a gradual decline in* H. pylori* eradication rates [6]. The drug resistance rates of* H. pylori* worldwide were [7] metronidazole 30.5~75.02% (average 47.22%), clarithromycin 5.46~30.8% (average 19.74%), and amoxicillin 2~40.87% (average 14.67%). In addition, a large number and a long-term use of antibiotics can cause gastrointestinal dysfunction and gastrointestinal flora imbalance and other adverse effects [8].

### 1.2. Introduction of Intervention

*Coptis *is the rhizome of Ranunculaceae plants* Coptis chinensis.* According to the theory of Chinese medicine, the character of* Coptis *is cold and its taste is bitter. Its functions are clearing away heat and eliminating dampness as well as purging fire and toxic material [9]. Berberine is the main active component of* Coptis*. Previous researches have found that* Coptis* has an inhibitory effect on* H. pylori*, and its antibacterial ring reached to 25 mm [10]. In addition, pharmacology has proved that berberine is capable of regulating the level of intracellular Ca^2+^, participating in the regulation of cytokines, inhibiting the formation of free radicals, and scavenging free radicals, therefore inhibiting mucosal inflammation and protecting the role of mucous membrane. What is more, berberine in the gastric mucosa can be quickly absorbed and maintain a high local drug concentration for a long time, which are conducive to its effective role in the gastric mucosa [11]. An increasing number of clinical controlled studies have found that berberine combined with triple therapy could improve the eradication rates of* H. pylori* and reduce the incidence of adverse effects, but there is no systematic review of it. Therefore, based on the extensive collection of literature, we use meta-analysis to compare the* H. pylori* eradication rates and adverse effects incidence between berberine combined with triple therapy group and triple therapy without berberine.

## 2. Methods

### 2.1. Eligibility Criteria

*Types of Studies.* We pooled all randomized controlled trials (RCTs) that compared* H. pylori* eradication rates as well as adverse effects incidence between berberine combined with triple therapy group and triple therapy group, regardless of the period of treatment.

*Types of Participants. *The participants included were* H. pylori *infected patients, regardless of age and gender. The diagnostic criteria simultaneously meet one or more of the following items showing positive results: rapid urease test (RUT), 13C-urea breath test (13CBUT), 14C-urea breath test (14CBUT), or histology.

*Types of Interventions.* The patients of experimental group were given berberine combined with triple therapy. Patients of control group were treated with triple therapy. The period was not considered.

*Types of Outcome Measure. *The primary outcome measure was eradication rate of* H. pylori *after treatment, which was simultaneously confirmed by one or more of the following items showing negative results: RUT, 13CBUT, 14CBUT, or histology. The secondary outcome measure was incidence of adverse effects (overall and specific, including nausea, diarrhea, vomiting, and abdominal distention).

### 2.2. Search Strategy

PubMed, MEDLINE, EMBASE, Cochrane Library, and Chinese databases including China National Knowledge Infrastructure (CNKI), Wanfang data, Chinese Technology Journal Full-text Database (VIP), and China biomedical literature database (CBM) were searched to obtain the eligible studies published up to October 30, 2017. Various combinations of Medical Subject Headings and non-MeSH terms were used. The following terms including “Berberine”, “Umbellatine”, “Berbines”, “Huang Lian Su”, “Xiao Bo Jian”, “*Helicobacter pylori*”, “*Campylobacter pylori*”, “*H Pylori*”, ‘*H. Pylori'*, “triple therapy”, and “triple treatment” were searched individually or in combination. Any language, populations, or country restrictions were not applied. The specific search strategy was shown as follows (taking PubMed as an example):

Berberine[MeSH] OR Umbellatine[Title/Abstract] OR Berbines[Title/Abstract] OR Huang Lian Su[Title/Abstract] OR Xiao Bo Jian[Title/Abstract]
*Helicobacter pylori*[MeSH] OR* Campylobacter pylori*[Title/Abstract] OR* H Pylori*[Title/Abstract] OR* H. Pylori*[Title/Abstract]
triple therapy[Title/Abstract] OR triple treatment[Title/Abstract]
(1) AND (2) AND (3).

### 2.3. Study Selection and Data Extraction

The pooled studies were RCTs. We excluded those articles meeting one of the following items: (i) the duplicates, (ii) the participants who did not meet the diagnosis criteria of* H. pylori* infection or the diagnosis criteria which were unknown, (iii) not RCT studies, (iv) the studies in which the experimental participants did not receive berberine combined with triple therapy as the primary intervention, (v) the intervention that contained any other traditional Chinese medicine therapy, and (vi) incomplete data which was required. Studies whether eligible were evaluated by two reviewers (HB and HC) independently. A third reviewer (XJ) was required when there was any disagreement during the articles inclusion. A Preferred Reporting Items for Systematic Reviews and Meta-Analyses (PRISMA) flow chart exhibited the process of studies screening. Two reviewers (GC and KJ) conducted data extraction independently according to predefined criteria. All reviewers discussed when disagreement existed during the data extraction. The following items were extracted: the first author, the year of publication,* H. pylori* diagnostic criteria,* H. pylori* eradication criteria, the patient characteristics, the sample size of study, the eradication rate, and incidence of adverse effects.

### 2.4. Risk of Bias Assessment

We evaluated the risk of bias of every included article according to the Cochrane Handbook for Systematic Reviews of Interventions. The methodological quality was assessed from the following seven aspects: random sequence generation, allocation concealment, blinding of participants and personnel, blinding of outcome assessments, incomplete outcome data, selective reporting, and other bias. The risks were categorized as low, high, or unclear.

### 2.5. Data Synthesis and Analysis

We used RevMan 5.2 software to analyze the related research indicators. Eradication rate of* H. pylori* was expressed by risk ratios (RR) and 95% CI, while incidence of adverse effects was expressed by odds ratio (OR) and 95% CI. I^2^ test was used to detect the heterogeneity of included studies. I^2^ > 50% indicated a significant heterogeneity, and under this circumstance, subgroup-stratified analysis, influence analysis, and meta-regression analysis would be performed. The choice of random effect model or fixed effect model depended on whether there existed heterogeneity or not. The potential publication bias was analyzed by Egger or Begg test when studies were sufficient (n≥10). If publication bias existed, shearing and mending method would be used to evaluate the impact of publication bias for the results.

### 2.6. Trial Sequential Analysis

The repeated significance test of known cumulative data will increase the overall risk of I errors in a single RCT [12, 13]. To solve it, statistical monitoring boundary can be used to evaluate if a single test can be terminated in advance, because a small enough *P* value can show the expected effect or uselessness. By combining information size estimation with an adjusted threshold for statistical significance in the cumulative meta-analysis, trial sequential analysis (TSA) was introduced to assess the risk of I errors. The latter is called the test sequence monitoring boundary, adjusting the confidence interval and reducing the I error [12, 13]. Use Obrien-Fleming alpha spending function to calculate the superiority or inferiority or uselessness. When the cumulative Z-curve passes through the sequential monitoring boundary, it may have reached sufficient level of evidence for the expected intervention effect and does not require further tests [14]. If the Z-curve does not exceed any boundary and has not reached the required amount of information, the evidence for conclusion is not sufficient [15].

## 3. Results

### 3.1. Description of Studies

**Figure 1:**
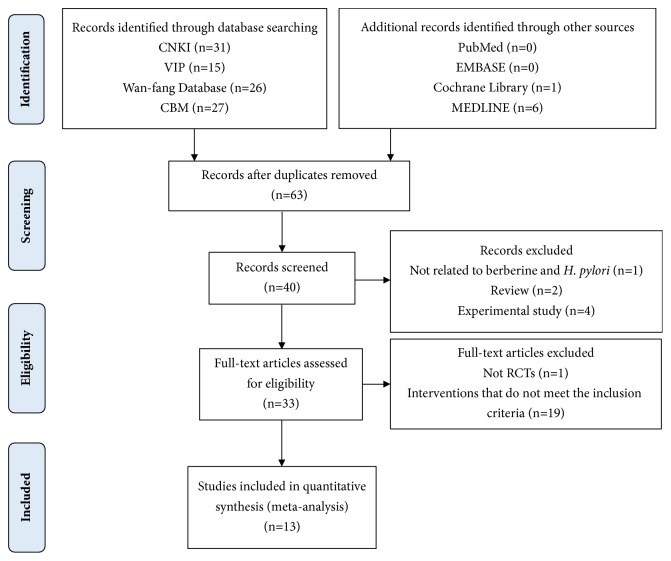
Study selection flow diagram.

A total of 106 articles were initially obtained through our search strategy. 40 of them were considered in the first stage of screening after eliminating 63 duplicated studies. The title and abstract of these 40 literatures were extracted and 7 articles were excluded because of type and unrelated subject. Of the remaining 33 articles, there were 19 articles of which interventions do not meet the inclusion criteria, and one was retrospective study. Finally, 13 studies meeting the inclusion criteria were utilized for further data extraction. The PRISMA flow diagram (Figure 1) presented the detailed process of screening.

### 3.2. Study Characteristics

The primary information of 13 pooled studies [16–28] was presented in Table 1. 2048 participants were included, 1036 of whom were from berberine combined with triple therapy group while 1012 were treated only with triple therapy. 7 studies used UBT [17, 19–21, 25, 27, 28], 2 used RUT [24, 26], and 2 used histology combined with RUT [16, 22] as the methods of both* H. pylori* diagnosis and eradication judgment. 1 study [23] used RUT(+) as* H. pylori* infection criteria and RUT(-) accompanied with 14CUBT(-) as eradicated judgment. 1 study [18] adopted RUT(+) accompanied with histology(+) as diagnostic criteria while considered participants simultaneously meeting histology(-), RUT(-), and 13CUBT(-) were uninfected with* H. pylori*. The average age ranged from 39.8 to 62.25 years. 8 studies used berberine [17, 20–23, 25, 26, 28], 2 used berberine hydrochloride [16, 27], and 3 used compound berberine tablet [18, 19, 24]. The duration of treatment lasted 7 days or 14 days. The primary adverse effects were nausea, vomiting, abnormal taste, and abdominal distension.

### 3.3. Risk of Bias

**Figure 2:**
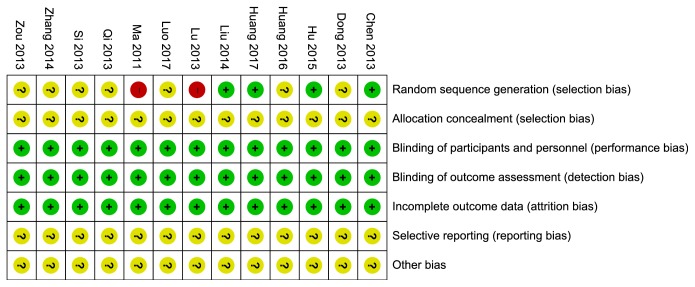
Risk of bias summary of included studies.

4 trials [23, 25, 27, 28] used random number table and were judged as low risk. 2 studies [17, 19] were identified as high risk in random sequence generation because they divided participants into groups according to whether applying berberine. The rest just only claimed random sequence without more detailed method which were judged as unclear risk of bias. Allocation concealment was unclear for not mentioned. As the outcome was objective and blinding method would exert little bias for the results, blinding of participants and personnel as well as outcome assessment was identified as low risk of all pooled articles. The data of all pooled studies were complete. As each included study's protocol was unavailable, selective reporting could not be judged. Other bias was judged as unclear risk of all pooled studies (see Figure 2).

### 3.4. Eradication Rates

**Figure 3:**
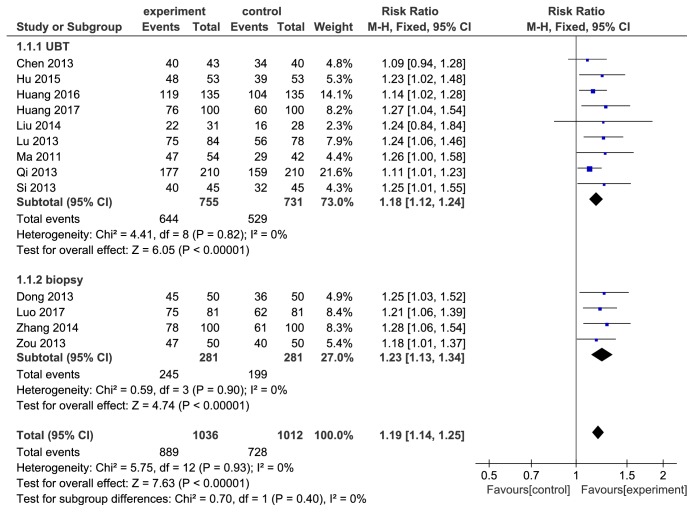
Forest plot of comparison of* H. pylori* eradication rates in UBT and biopsy subgroup between berberine plus triple therapy and triple therapy.

All 13 articles reported the* H. pylori* eradication rates. However, their diagnostic and eradicated criteria of* H. pylori* were different. Although there were only minor differences in accuracy among RUT, UBT, histology for* H. pylori* positive prediction [29], the negative prediction accuracy is not always the same. RUT and histology examination, both of which are biopsy, are influenced by* H. pylori* uneven distribution and more prone to present false negative than UBT which reflects the infectious status of the whole stomach [30–33]. Therefore, to assure the reliability of results, it is necessary to divide UBT with two other methods into two layers according to whether UBT was applied in eradicated criteria when conducting analysis of eradication rates. 9 studies [17–21, 23, 25, 27, 28] used UBT to detect whether* H. pylori* was eradicated, which were pooled in UBT subgroup while 4 studies [16, 22, 24, 26] pooled in biopsy subgroup adopted RUT alone or in combination with histology. There were no significant heterogeneity in UBT subgroup (*P* = 0.82, I2=0%) and biopsy subgroup (*P* = 0.40, I2=0%). As Figure 3 showed, the eradication rates of berberine combined with triple therapy were higher than the control group (UBT subgroup: RR=1.18, 95%CI=(1.12,1.24), *P* < 0.00001, biopsy subgroup: RR=1.23, 95%CI=(1.13,1.34), *P* < 0.00001).

### 3.5. Eradication Rates Subanalysis Based on Course

**Figure 4:**
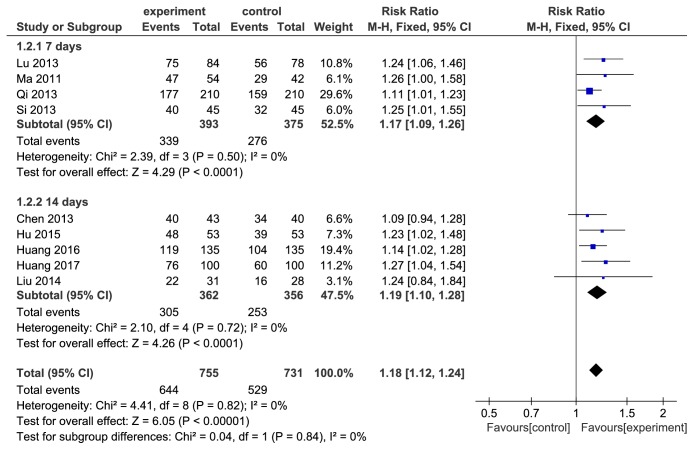
Forest plot subgroup analysis based on duration in UBT subgroup of comparison of* H. pylori *eradication rates between berberine plus triple therapy and triple therapy.

**Figure 5:**
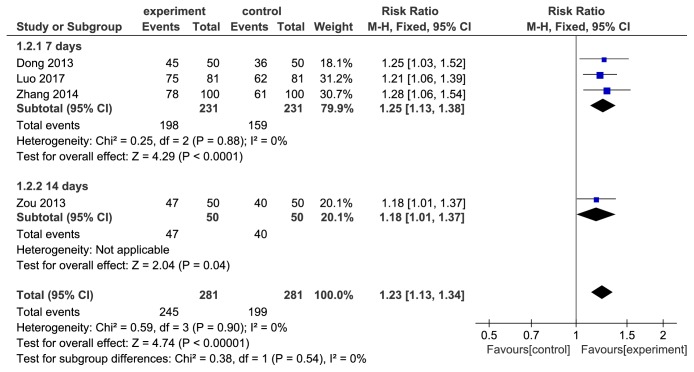
Forest plot subgroup analysis based on duration in biopsy subgroup of comparison of* H. pylori* eradication rates between berberine plus triple therapy and triple therapy.

Subgroup analysis was performed based on the course of treatment. In UBT subgroup, 4 studies [17–20] of berberine combined with triple therapy compared the eradication rates with triple therapy for 7 days, revealing significant effects favoring berberine intervention (RR=1.17, 95%CI(1.09,1.26), *P* < 0.0001) (Figure 4). 5 studies [21, 23, 25, 27, 28] compared the eradication rates on berberine plus triple therapy with triple therapy alone for 14 days in UBT subgroup, which revealed that berberine combined with triple therapy improved the eradication rate (RR=1.19, 95%CI(1.10,1.28), *P* < 0.0001) (Figure 4). In biopsy subgroup, there were 3 studies [16, 22, 24] with treatment for 7 days and 1 study [26] for 14 days. The eradication rates of berberine combined with triple therapy were higher compared to the control group in biopsy subgroup (7 days of treatment: RR=1.25, 95%CI(1.13,1.38), *P* < 0.00001, 14 days of treatment: RR=1.18, 95%CI(1.10,1.27), *P* < 0.00001) (Figure 5).

### 3.6. Adverse Effects and Subanalysis Based on Symptoms

**Figure 6:**
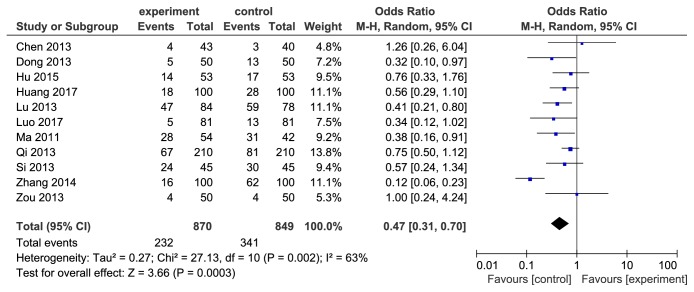
Forest map of comparison of adverse effects incidence between berberine plus triple therapy and triple therapy before excluding the article with heterogeneity.

**Figure 7:**
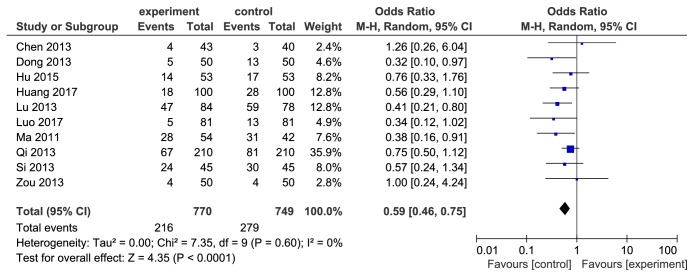
Forest map of comparison of adverse effects incidence between berberine plus triple therapy and triple therapy after excluding the article with heterogeneity.

**Figure 8:**
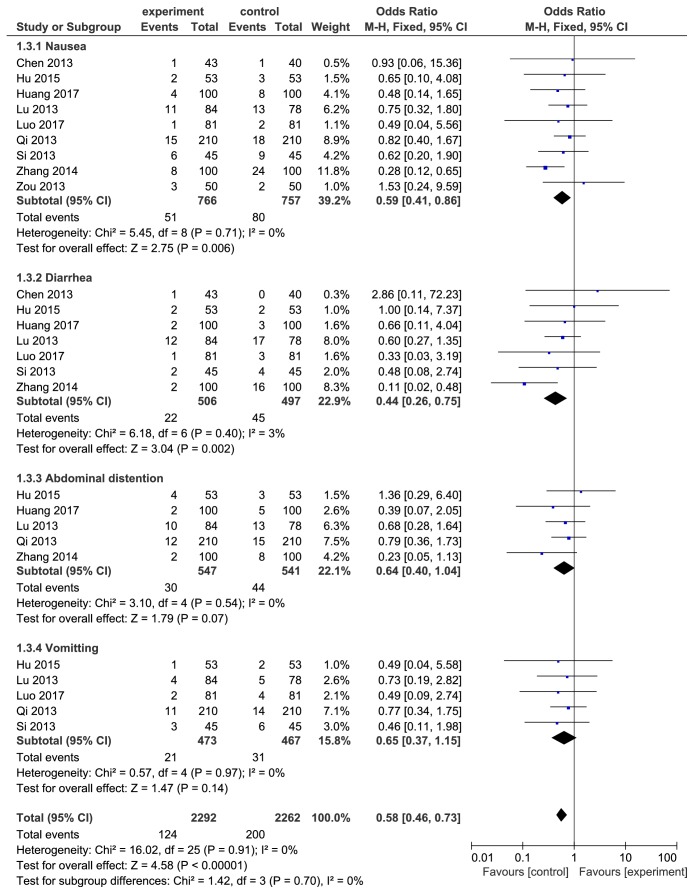
Effect of berberine combined with triple therapy versus triple therapy on the incidence of primary adverse effects including nausea, diarrhea, abdominal distention, and vomiting.

11 included studies [16–20, 22–26, 28] reported the adverse effects incidence. Since the* H. pylori* detection methods in primary diagnosis and after therapy did not affect the incidence of adverse effects, it was not necessary to carry out a subgroup analysis of adverse effects incidence based on diagnostic and eradicated judgment methods. We performed a heterogeneity test and detected remarkable heterogeneity of the 11 pooled studies (I^2^=63%, *P* = 0.0003, see Figure 6). Zhang's [22] study was found as the source of heterogeneity after we conducted influence analysis. The literature was excluded and the result was remerged again. The adverse effects incidence of berberine combined with triple therapy was lower than the control group (OR=0.59, 95%CI(0.46, 0.75), *P* < 0.0001, see Figure 7). Adverse symptoms including nausea, diarrhea, abdominal distention, and vomiting were subanalyzed (see Figure 8). Incidence of nausea (OR=0.59, 95%CI(0.41, 0.86), *P* < 0.05) and diarrhea (OR=0.41, 95%CI(0.24, 0.71), *P* < 0.05) was lower in berberine combined with triple therapy group, while abdominal distention (OR=0.64, 95%CI(0.40,1.04), *P* > 0.05) and vomiting (OR=0.65, 95%CI(0.37, 1.15), *P* > 0.05) had no remarkable difference between two groups.

### 3.7. Publication Bias

**Figure 9:**
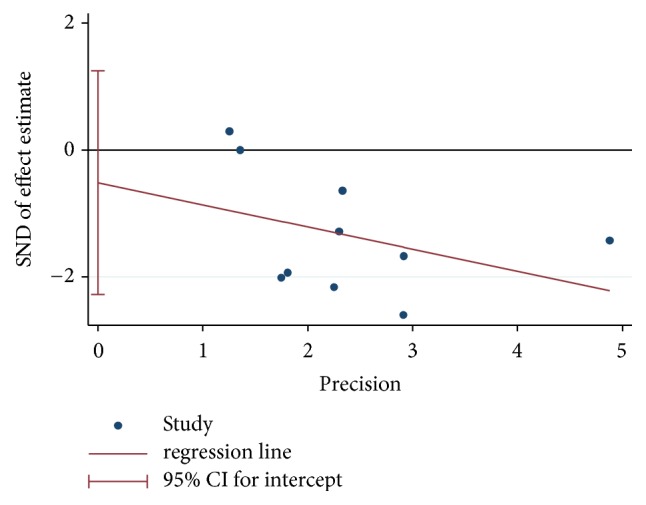
Egger's funnel plot of adverse effects incidence rate.

Funnel plot analysis of* H. pylori* eradication rates could not be completed because of insufficient studies (n<10) in UBT subgroup or biopsy subgroup. As for the adverse effects incidence, Egger's test did not detect the publication bias (*P* = 0.52, see Figure 9).

### 3.8. Trial Sequential Analysis

We conduct TSA of eradication rates and adverse effects incidence.

**Figure 10:**
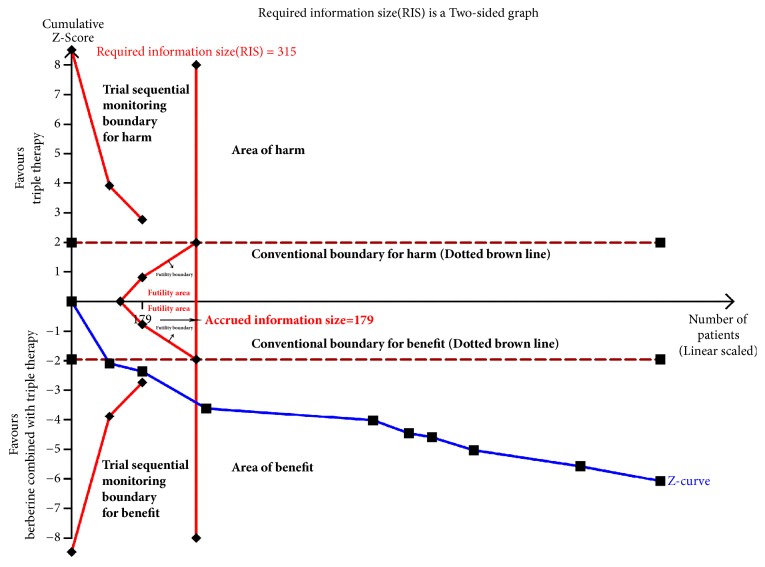
Trial sequential analysis of* H. pylori* eradication rates in UBT subgroup.

In UBT subgroup, eradication rates of 9 trials were included in the meta-analysis. A total of 1486 cases were included in the study, and the required information size (RIS) was 315 calculated based on the following statistical indicators: I error probability (*α* = 0.05), type II error probability (*β* = 0.2), relative risk reduction (RRR=-17.82%), and the incidence in control arm (Pc= 72.4%, derived from the meta-analysis data). The TSA results showed that the cumulative value of Z went across the conventional boundary value, trial sequential monitoring boundary for benefit, and RIS (see Figure 10), suggesting the trials were enough and further trials were not likely to alter the conclusion.

**Figure 11:**
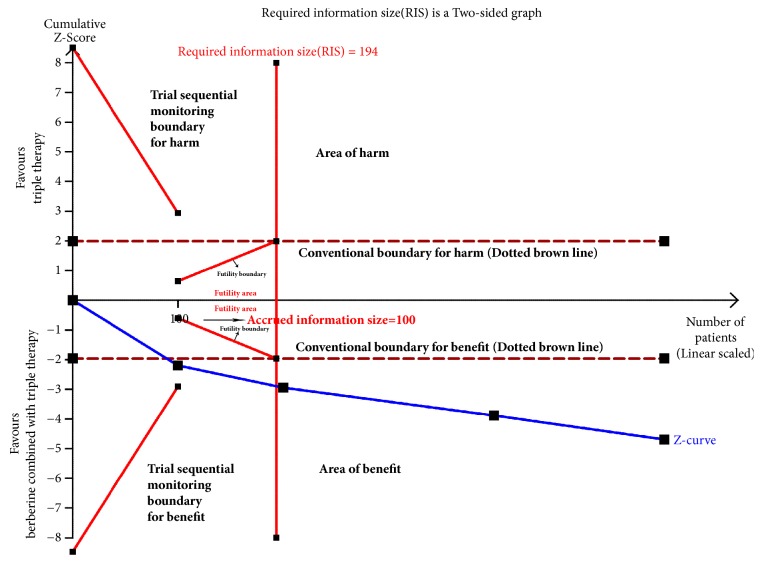
Trial sequential analysis of* H. pylori* eradication rates in biopsy group.

In biopsy subgroup, eradication rates of 4 trials were included in the meta-analysis. A total of 444 cases were included in the study, and the RIS was 194 calculated based on type I error (*α* = 0.05), type II error (*β* = 0.2), relative risk reduction (RRR=-23.16%), and the incidence in control arm (Pc= 70.8%, derived from the meta-analysis data). The TSA results illustrated that the Z-curve went across the conventional boundary value, trial sequential monitoring boundary for benefit, and reach RIS (see Figure 11), suggesting the trials were enough to draw the reliable conclusion.

**Figure 12:**
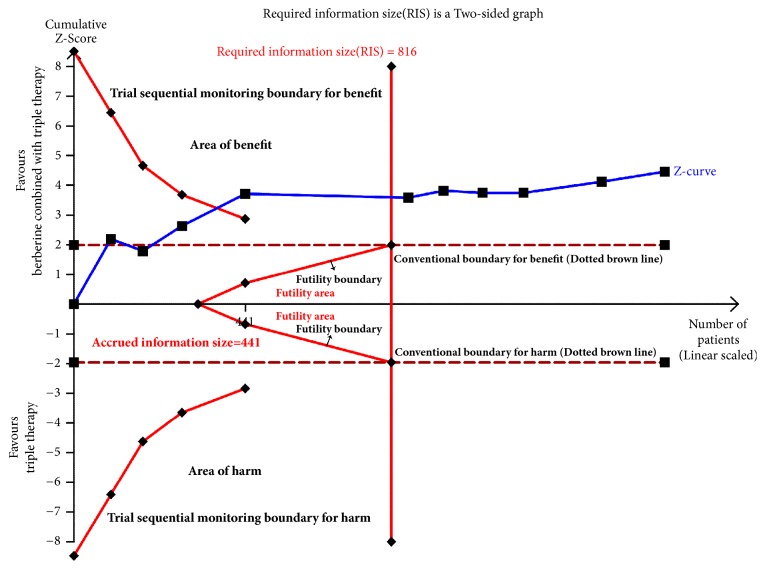
Trial sequential analysis of adverse effects incidence.

Adverse effects incidence of 10 trials was included in the meta-analysis. A total of 1519 cases were included in the study. The RIS was 816 calculated based on type I error probability (*α* = 0.05), type II error probability (*β* = 0.2), relative risk reduction (RRR=24.73%), and incidence of adverse effects in control group (Pc = 37.2%, derived from the meta-analysis data). The cumulative value of Z went across the traditional boundary value, TSA boundary, and RIS (see Figure 12), which means the pooled sample size of adverse effects incidence was adequate to get a stable conclusion.

## 4. Discussion

### 4.1. Summary of Main Results

Metronidazole, clarithromycin, and amoxicillin are the most commonly used antibiotics in the first-line regimen for the treatment of* H. pylori* infection, whereas, due to the widespread use of these antibiotics, the drug resistance of* H. pylori* problem is increasingly severe [34]. Berberine has a broad-spectrum antibacterial effect and has been widely used for intestinal infectious diseases clinically. Increasing clinical studies have found that berberine alone or with triple therapy could improve the eradication rates of* H. pylori* and the cure rates of gastritis and gastric ulcer. Thus, we conducted a meta-analysis to further prove its effectiveness. Berberine combined with triple therapy could increase the* H. pylori* eradication rates and lower the total number of adverse effects. Specifically, nausea and diarrhea incidence were remarkably lower in berberine combined with triple therapy group. TSA of both* H. pylori* eradication rates and adverse effects incidence suggested that the trials were enough to draw the reliable conclusion.

### 4.2. The Effectiveness of Berberine in H. pylori Eradication

The results have revealed the effectiveness that berberine combined with triple therapy could increase the* H. pylori* eradication rates no matter measured by UBT or biopsy. And, it is worth mentioning that the* H. pylori* eradication results in UBT subgroup are more reliable than biopsy subgroup. In UBT subgroup, urea labelled with either 14C or 13C is hydrolyzed by* H. pylori* urease enzyme and the labelled carbon dioxide is exhaled in the breath which can be used as an indicator of* H. pylori* colonization [35]. UBT is free of* H. pylori* uneven distribution because the distribution of urea throughout the stomach prevents sampling error [36]. However, the negative prediction of biopsy can be influenced by* H. pylori* uneven distribution in stomach when sampling and is more prone to present false negative [32, 33], which may overestimate the eradication rates. Therefore, it was more reliable to judge the effectiveness of berberine in* H. pylori* eradication based on the eradication results of UBT subgroup.

### 4.3. Mechanism of Berberine in Relieving Nausea and Diarrhea

The high concentration of antibacterial drugs in the digestive tract during the triple therapy treatment can increase the chance of intestinal flora imbalance, leading to reduction of carbohydrate metabolism or even double infection and causing antibiotic-associated diarrhea [37, 38]. Besides, clarithromycin, a kind of macrolide antibiotic in triple therapy regimen, has a motilin-like effect that it can accelerate gastrointestinal peristalsis and shorten transit time, leading to diarrhea, nausea, and so forth [39, 40]. Berberine is capable of regulating the intestinal flora, significantly promoting the growth of probiotics and inhibiting the growth of pathogens, which can relieve antibiotic-associated diarrhea [41]. In addition, it can affect the excitation contraction coupling of intestinal smooth muscle and inhibit the contraction of smooth muscle cells by inhibiting the increase of calcium concentration in intestinal smooth muscle cells, which is important to exerting the effect of nausea and diarrhea relieving [42].

### 4.4. Limitations

Several limitations may influence the validity of our research. Firstly, all of the pooled studies were not registered and protocols were unavailable. Secondly, allocation concealment of all studies was unknown which may affect the authenticity of the results. Thirdly, enslaved to the accuracy of* H. pylori *diagnostic and eradicated methods, our results may be modified by* H. pylori* false positive and false negative. Fourthly, none of the studies involved patients from any other countries or areas except China.

## 5. Conclusion

Although there existed some limitations of our research, it also provided an evidence that berberine combined with triple therapy has benefits for improving the* H. pylori *eradication rates and reducing overall therapy-related adverse effects incidence, particularly nausea and diarrhea. For overall low methodological quality of included literature, further studies should be strictly designed and conducted in multicenters.

## Figures and Tables

**Table 1:** Characteristics of included studies.

First author, year	*H. pylori* diagnostic criteria	*H. pylori* eradicated criteria	Accompany disease	Sample size	Mean age (years)		Treatment	Control	Duration of treatment (d)	Adverse effects
				(T/C)						
					T	C				
Ma 2011	13CUBT(+) RUT (+)	13CUBT(-) (4 wk later)	Peptic ulcer or Chronic gastritis	96(54/42)	40.94		CBT (4 tablets/time tid) +Control	C(500mg bid) A(1000mg bid) R(10mg bid)	7	①②③④⑤⑦

Si 2013	13CUBT(+) RUT (+)	13CUBT(-) RUT(-) (4 wk later)	Peptic ulcer	90(45/45)	43.5		B (0.4g tid) +Control	CH(500mg bid) A(100mg bid) O(20mg bid)	7	①②④⑤⑦

Hu 2015	13CUBT(+)	13CUBT (-) (4 wk later)	Peptic ulcer or Chronic gastritis	106(53/53)	49.45		B (0.5g tid) +Control	CH(400mg bid) A(1g bid) O(15mg bid)	14	①②④⑤⑦

Qi 2013	14CUBT(+)	14CUBT(-) (2 wk later)	Not mentioned	420(210/210)	44.2		B (0.3mg tid) +Control	C(0.25g bid) A(1g bid) R(20mg qd)	7	①②③④⑤

Huang2016	14CUBT(+)	14CUBT(-) (2 wk later)	Peptic ulcer	270(135/135)	46.2		B (300mg tid) +Control	C(500mg bid) A(1000mg bid) Or(20mg bid)	14	Not mentioned

Chen 2013	14CUBT(+)	14CUBT(-) (4 wk later)	Not mentioned	265(140/125)	55		B (0.3g tid) +Control	C(500mg bid) A(1000mg bid) O(20mg bid)	14	①②⑤⑫⑭

Liu 2014	14CUBT(+)	14CUBT(-)	Chronic atrophic gastritis	83(43/40)	62.25		BH (60mg bid) +Control	C(500mg bid) A(1000mg bid) O(20mg bid)	14	①②⑩⑬

Huang2017	RUT(+)	RUT(-) 14 CUBT(-) (4 wk later)	Peptic ulcer	200(100/100)	46.2		B (300mg tid) +Control	C(0.5g bid) A(1g bid) O(20mg bid)	14	①④⑤⑨⑭

Lu 2013	Histology(+) RUT(+)	Histology(-) RUT(-) 13CUBT(-) (4 wk later)	Peptic ulcer or chronic gastritis	162(84/78)	40.87		CBT (4 tablets/time tid) +Control	C(500mg bid) A(1000mg bid) R(10mg bid)	7	①②④⑤⑦

Dong 2013	Histology(+) RUT(+)	Histology(-) RUT(-) (4 wk later)	Not mentioned	100(50/50)	39.8		BH (120mg tid) +Control	C(0.5g bid) A(1g bid) R(10mg bid)	7	①②③④⑤⑥

Zhang 2014	Histology(+) RUT(+)	Histology(-) RUT(-)	Not mentioned	200(100/100)	43.3		B (120mg tid) +Control	C(0.5g bid) A(0.5g tid) R(20mg bid)	7	①④⑤⑧

Luo 2017	RUT(+)	RUT(-) (4 wk later)	Peptic ulcer	162(81/81)	42.14		CBT (30mg tid) +Control	C(0.5g bid) A(0.5g bid) O(20mg bid)	7	①②⑤⑩⑮

Zou 2013	RUT(+)	RUT(-) (4 wk later)	Peptic ulcer	100(50/50)	47.75		B (0.3g tid) +Control	C(500mg bid) A(1000mg bid) O(20mg bid)	14	①⑪

①nausea, ②vomiting, ③abnormal taste, ④abdominal distension, ⑤diarrhea, ⑥anorexia, ⑦poor appetite, ⑧acid reflux, ⑨constipation, ⑩liver function injury, ⑪upper abdominal discomfort, ⑫rash, ⑬leukocyte reduction, ⑭dizzy, and ⑮abdominal pain.

UBT: urea breath test; RUT: rapid urease test; BH: berberine hydrochloride; B: berberine; CBT: compound berberine tablet; C: clarithromycin; A: amoxicillin; R: rabeprazole; O: omeprazole; CH: clarithromycin hydrochloride

## Data Availability

The data used to support the findings of this study are included within the supplementary information files.

## Supplementary Materials

Supplementary 1 File S1: Extracted data and meta-analysis outcomes.

Supplementary 2 File S2: Egger's funnel plot of adverse effects incidence rate.

Supplementary 3 File S3: Trial sequential analysis of adverse effects incidence.

Supplementary 4 File S4: Trial sequential analysis of* H. pylori* eradication rates in biopsy group.

Supplementary 5 File S5: Trial sequential analysis of* H. pylori* eradication rates in UBT subgroup.
